# A New Architecture Based on IoT and Machine Learning Paradigms in Photovoltaic Systems to Nowcast Output Energy

**DOI:** 10.3390/s20154224

**Published:** 2020-07-29

**Authors:** Guillermo Almonacid-Olleros, Gabino Almonacid, Juan Ignacio Fernandez-Carrasco, Macarena Espinilla-Estevez, Javier Medina-Quero

**Affiliations:** 1 Department of Electronic Engineering, Campus Las Lagunillas, 23071 Jaén, Spain; gao00003@red.ujaen.es (G.A.-O.); jifernan@ujaen.es (J.I.F.-C.); 2 Department of Computer Science, Campus Las Lagunillas, 23071 Jaén, Spain; mestevez@ujaen.es (M.E.-E.); jmquero@ujaen.es (J.M.-Q.)

**Keywords:** photovoltaic systems, nowcasting energy generation, temporal windows

## Abstract

The classic models used to predict the behavior of photovoltaic systems, which are based on the physical process of the solar cell, are limited to defining the analytical equation to obtain its electrical parameter. In this paper, we evaluate several machine learning models to nowcast the behavior and energy production of a photovoltaic (PV) system in conjunction with ambient data provided by IoT environmental devices. We have evaluated the estimation of output power generation by human-crafted features with multiple temporal windows and deep learning approaches to obtain comparative results regarding the analytical models of PV systems in terms of error metrics and learning time. The ambient data and ground truth of energy production have been collected in a photovoltaic system with IoT capabilities developed within the Opera Digital Platform under the UniVer Project, which has been deployed for 20 years in the Campus of the University of Jaén (Spain). Machine learning models offer improved results compared with the state-of-the-art analytical model, with significant differences in learning time and performance. The use of multiple temporal windows is shown as a suitable tool for modeling temporal features to improve performance.

## 1. Introduction

Currently, photovoltaic (PV) power generation has been shown to be a successful technology with a remarkable level of maturity with more than 500 GW of solar photovoltaic (PV) power installed all over the world at the end of 2018, in some cases running for several years, and with a forecast of 1 TW of total power being generated by 2022, most of it in large PV plants. The management of the operation and maintenance (O&M) of these systems is a relevant research field for the solar PV industry [1,2].

Data represent a key asset in this PV management area, since they enable us to model the standard behavior of the system and to monitor its performance compared with the expected output determined by the model. This monitoring, when applied promptly and comprehensively, taking account of all the factors that may impact performance, enables early damage and fault detection, which then allows operation and maintenance actions to maximize the up-time and efficiency of PV plants.

Traditionally, approximate analytical expressions based on the physical laws and the electrical parameters of the solar cells, together with the engineering data of the devices that conform the PV system, have been used to build standard performance models. Leveraging the latest software advances in machine learning, a different approach can be taken by using regressors to build models, which learn from data on the actual behavior of the system during a relevant period of time and use the time series prediction to monitor performance. Machine learning approaches bring the advantage of modeling independently from the deployment and configuration parameters of the PV system, which are strongly affected by location and environmental conditions.

This work presents an important extension of the proposal [3], where two deep learning models showed a better performance in forecasting energy generation with regard to standard analytical models [4,5]. The main contribution of this work is evaluating in further detail the capabilities of data-driven models for nowcasting the energy generation of photovoltaic systems from ambient sensor information. In this way, two main data-driven approaches are evaluated: (i) human-crafted features which are computed by means of multiple temporal windows and (ii) deep learning models with automatic feature extraction and learning. Several configurations of segmentation and aggregation by means of temporal windows have been proposed showing an improvement in terms of performance and learning time. So, an important advance is made in this knowledge area through the use of machine learning techniques to make predictions about PV system consumption in order to check its status. In addition, an IoT module which collects photovoltaic data in real time within the Opera platform is described. The module has collected the evaluation data over 24 weeks, which are openly available to the scientific community.

The remainder of the paper is organized as follows: in Section 2, we detail the review of works related to our proposal; Section 3 describes the supporting infrastructure and IoT module for collecting real-time data within the Opera Digital Platform; Section 4 presents the methodology to develop data-driven nowcasting of PV system consumption; Section 5 introduces the results of the dataset collected by the Opera Digital Platform. Finally, conclusions and ongoing works are discussed in Section 6.

## 2. Related Works

PV systems are now considered a well-established technology for energy generation and have reached a significant maturity level. However, being relatively recent most of the systems have been running no more than 20 years [1,6]—means that there is not much experience in Operations and Maintenance (O&M). Most of the tasks and tools regarding O&M make little use of new information technologies such as big data, deep learning, business intelligence, etc. [7]. Up to now, the most common way to estimate the behavior of PV systems has been the use of classic models based on the physical process of the solar cell to define the analytical equation to obtain its electrical parameter [8]. There are many of these models with very different approaches, difficulty levels and results [4,9,10,11]. The main objective of these tools is to nowcast the electrical energy generated by the cells and also by the PV system.

Among all of these classic models, we have selected the Araujo model plus constant FF (FF: Fill Factor is a noteworthy solar cell figure regarding maximum power delivered vs. maximum current and maximum voltage of the cell; its upper limit is 1;) [4] to compare and evaluate the performance of PV systems with the performance estimated by our proposed machine learning model. Araujo is a standard PV model that combines enough accuracy with a very simple formulation [4,8]; additionally, it needs only a few variables to be measured: current and voltage of the cell, irradiation and ambient temperature [5].

Nevertheless, to obtain output energy using any of these classic models it is necessary to know a large number of parameters and specifications of the PV generator in question: technical specs, topology of the generator, location etc. One of the main advantages of our machine learning-based proposal is the ability to nowcast all of these parameters and specs independently, and hence enabling easier and more efficient PV deployment and customization.

Recently, several works regarding the use of new technologies to monitor and nowcast PV system behavior have been presented. However, none of them have been used in or have produced—a usable O&M management system [7,12,13,14]. A previous work related to a O&M analytics platform was presented in [15]. The use of new information technologies in O&M management in the renewables sector has, up to now, been restricted to a few large and expensive platforms developed by companies to use in utility-scale generator power plants [16,17].

Regarding the collection of operating data to monitor PV systems, it is traditionally carried out with wired sensor data acquisition systems, which are sometimes expensive, allow little flexibility and have limited cloud connectivity. Recently, several works on the new concept of using IoT connectivity in monitoring the behavior of PV systems have been presented [12,14,18]. Incorporating these sensors in a comprehensive O&M management tool has allowed us to develop a highly versatile and easy-operation data collection system with wireless sensors, which offers great advantages as regards ease of use, cost efficiency and standardization of data capture [13,19,20].

Several proposals based on the IoT paradigm in photovoltaic systems have been presented in the relevant literature. In [21], a literature review of IoT energy platforms aimed at end users is presented, where platform selection, new energy platform construction and, finally, platform comparison are considered. In [22], the design and implementation of an IoT-based solar monitoring system for city-wide, large-scale, and distributed solar facilities in smart cities was presented. In [23], a solar tracking system enabling increased efficiency of photovoltaic systems was proposed. The proposed system executes a tracking algorithm in the Firebase web service and allows the exchange of data with said service through a NodeMCU development board, which has an integrated Wi-Fi module. Finally, in [24], the use of IoT and machine learning paradigms for next-generation solar power plant monitoring systems was analyzed and discussed.

Regarding the use of IoT and machine learning paradigms for analyzing sensor data streams, there are techniques that have proven to be successful in other contexts. For example, evaluation of single and multiple windows to segment and fuse temporal information from sensor data streams [25,26], whose window size can be imbalanced [27,28] to aggregate data from shorter to longer terms, enriching the features of sensor streams.

On the other hand, the use of Deep Learning in temporal series has become a prolific research field [29]. Mainly, with the use of Long-Short Term Memory (LSTM) [30], which is a type of recurrent neural network that includes a memory and is designed to learn from sequence data, such as sequences of observations over time. LSTM is most widely used in natural language processing and speech recognition, can model temporal dependence between observations [31] and is suitable for prediction from sensor data [32]. LSTM has obtained encouraging results in several fields, such as activity recognition [28] or estimating building energy consumption [33]. Moreover, modeling spatial features in time series by means of Convolutional Neural Networks (CNNs) [31] qiu2017learning has achieved promising results in speech recognition [34] or gas classification [35], together with LSTM models [36].

## 3. IoT Module for Real-Time Data Collection in the Opera Digital Platform

In this section, we describe the IoT module for collecting the photovoltaic data in the Opera Digital Platform, which have been collected to nowcast output energy generation in the photovoltaic system.

**Figure 1:**
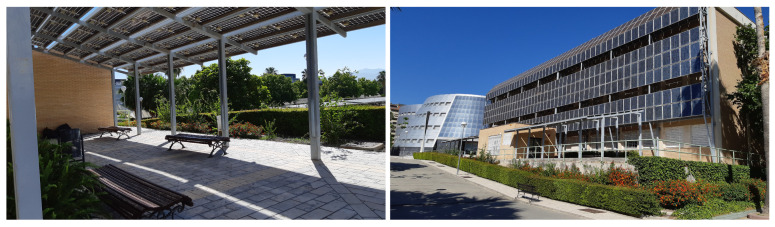
Two views of the photovoltaic (PV) generator of the UniVer Project. (**a**) PV pergola with semitransparent modules; (**b**) East view of the PV facade.

Opera Project is a digital platform developed by an interdisciplinary team, covering the areas of ICTs, PV and Electronic Technology, and has been designed to provide O&M management services for renewable energy installations [15]. This digital platform has been developed with the knowledge and the working data of the UniVer Project. This project see Figure 1 is a standard, medium-sized, grid-connected PV system that has been running for the last 20 years in the Campus of the University of Jaén [37]. The PV modules are made of 60 multicrystalline Si solar cells with 18.34% efficiency and a 156.75 × 156.75 mm${}^{2}$ surface. The PV generator is composed of 220 of these modules with a topology of 20 (serial) × 11 (parallel) and a total power of 59.4 kW at Standard Test Conditions (STCs; that is, 1000 W/m${}^{2}$ of normal irradiance onto cells, cell temperature of 25 ${}^{\circ }$C and AM1.5 solar spectrum).

The Opera Platform is now also managing the O&M of this PV system.

The main objective of the IoT-based PV system O&M optimization module, besides reducing costs, is to monitor the generated energy. Energy $E_{T}$ is the end product of every electric generator and is computed as the integral of instantaneous power *P* in a period of time T: $E_{T}=\int_{T}P\cdot dt$. Electric power output is the instantaneous variable to be measured by this data collection system and also targeted by the models to nowcast the behavior of PV systems, such as the one developed in this paper. This output mainly relies upon the entry product: solar irradiance *G* whose magnitude is defined by the square density of power incident on a surface measured in Watts per square meter ($W/m^{2}$). The temperature and the specs of the PV generator (PVG) are the other inputs for this data collection system monitoring the performance of the PVG.

Monitoring of the PVG must be done following the European Standard IEC 61724 [38]. In line with this, the variables that have been measured are shown in Table 1. From these measured data and with the nominal specs of the PVG at STC, we compute derived parameters and metrics regarding losses in energy performance, which is useful to evaluate the behavior of the PV system and very helpful for fault diagnosis and descriptive operation analysis, such as: (i) global irradiation on the PVG surface, (ii) net energy from the PVG in a period of time, (iii) performance ratio and (iv) yields and losses. All of them are well defined analytically and conceptually in [38] and their function, meaning and usefulness are also described in [39,40,41]. In this work, we have focused on the nowcasting of output power generation, which is straightforwardly related to the analytical metrics on the behavior of the PV system.

In order to collect environmental and energy generation information from the Opera Digital Platform in real time, we have developed and deployed a genuine integration of ambient and power supply sensors. This is composed of a set of sensors based on IoT technology connections and controlled by a microprocessor which uploads the data by wireless network. These sensors measure the working data of the PVG and the environmental variables shown in Table 1, needed to monitor and nowcast PVG operation in accordance with standard [42].

The central unit of the IoT module is an Arduino. It is a standard board device that includes, in addition to a μP, an input data conditioner, a communication network interface and other display interfaces. The IoT module is responsible for collecting the photovoltaic data to send the information to the cloud by means of an internet connection (i.e.: wired, WiFi or modem). The module is powered by standard power supply or by solar panel plus battery.

**Figure 2:**
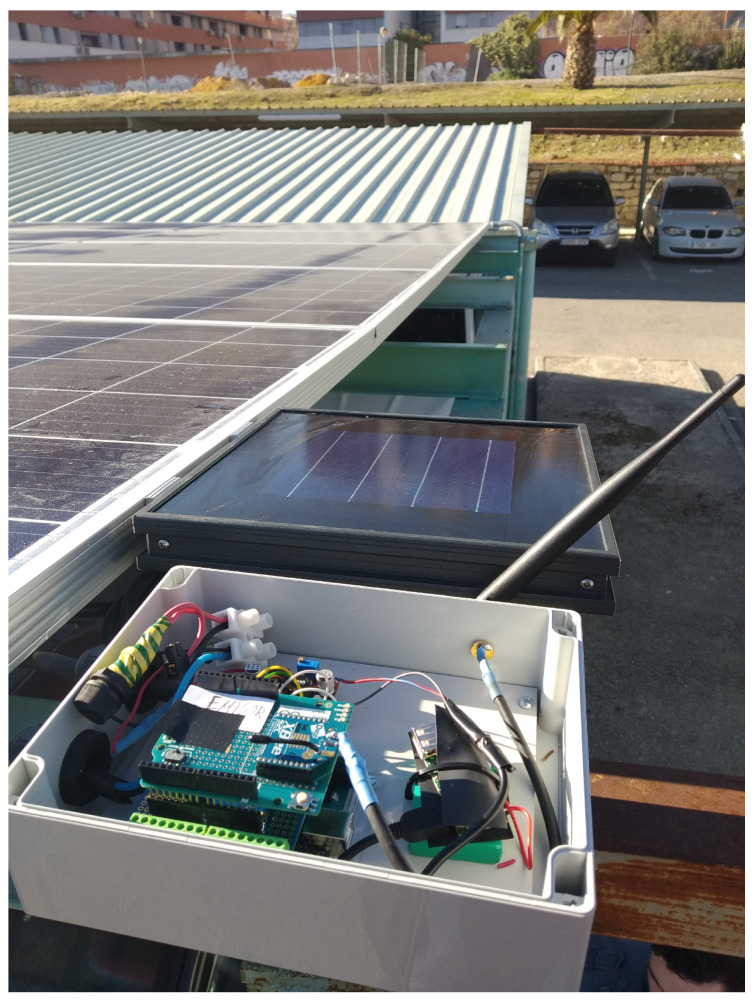
Radiation and temperature sensors unit.

The ambient sensors connected to the Arduino board detect: (i) solar irradiance, (ii) module temperature and (iii) ambient temperature. The irradiance sensor is a calibrated Si solar cell (calibration certificate from CIEMAT, the Spanish Research Centre in Energy, Environment and Tech.), with an analogical output from 0–5 V corresponding to an irradiance range from 0 to 1250 W/m${}^{2}$. The ambient and cells temperature sensors are 4-wire Pt100 Probes, also with an analogical output of 0–5 V, corresponding to a temperature range of −20 to 130 ${}^{\circ }$C. These two sensors, plus the corresponding interface circuitry, are included in a commercial unit made by Atersa S.L. (www.atersa.com), as we describe in Figure 2. The ambient sensors are placed close to the panels and are powered by their own solar mini-module. The ambient sensors send the measured data to the Arduino microprocessor using Zigbee protocol to enable direct wireless communication between the devices and the Arduino board [43] in open areas, which is inherent in the deployment of photovoltaic systems. We included the Zigbee connection since experimental results with other popular wireless technologies, such as Wi-Fi and Bluetooth, show that it is more energy efficient [44].

The PVG data measured by the IoT module are the instantaneous values of output voltage and intensity, which enable the computing of output power by multiplying output voltage and current intensity. This is possible since the data are instantaneous values; in this case, the output of the PVG is DC current, so this way to obtain power is also valid for mean values over a period of time. Alternatively, an output power sensor can be installed, such as a power meter or a grid analyzer, to get some redundancy in the measured data and, with the second device, some additional secondary electrical output parameters.

**Figure 3:**
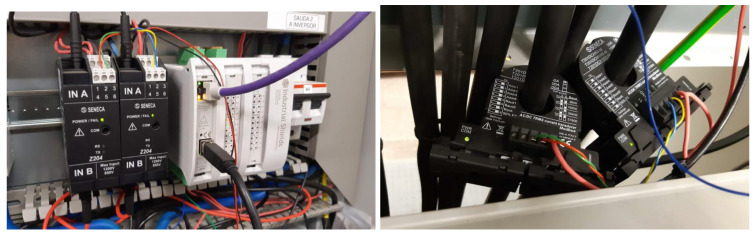
Current and voltage sensors and microprocessor unit. (**a**) Two voltage sensor devices for the two branch of the generator under study (black units) and the μP unit (white one); (**b**) Two current sensor, toroidal cores, for the two branch of the generator under study.

**Figure 4:**
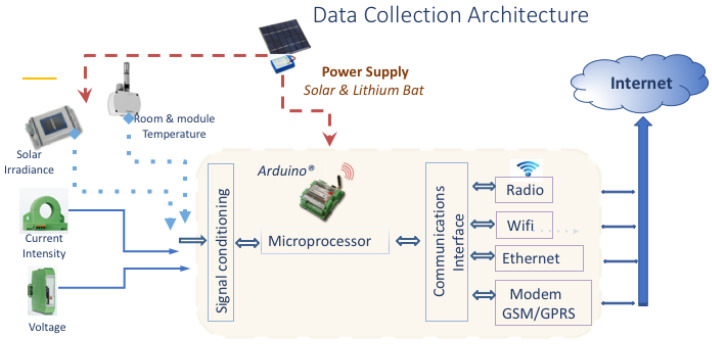
Architecture of the IoT module for collecting data in real-time.

Finally, in Figure 3 we show the voltage and current sensors, along with the microprocessor unit used to measure operation data of the UniVer Project PV generator. Figure 4 shows a schematic diagram of the data collection architecture.

## 4. Machine Learning Approaches to Nowcast Power Generation

In this section, we describe the methodology used for processing, segmenting and modeling the sensor data from the Opera PV System in order to nowcast output power generation from the ambient sensor information in real time.

As stated previously, several models are evaluated in this work. They are mainly grouped into: (i) human-crafted features and multiple temporal windows and (ii) deep learning for automatic feature extraction and learning. In the following sections, we detail: (first) basic segmentation with temporal sliding windows for sensor streams in a data-driven model; (second) modeling for human-crafted features and multiple temporal windows; and (third) deep learning approaches to nowcast output power generation of the Opera PV System.

### 4.1. Data-Driven Model to Nowcast Power Generation

Following a formal definition, a sensor *s* collects data in real time in the form of a pair $\bar{s_{i}}=\left\{s_{i},t_{i}\right\}$, where $s_{i}$ represents a given measurement and $t_{i}$ the time-stamp, respectively. Thus, the data stream of the sensor source *s* is defined by $\bar{S_{s}}=\left\{\bar{s_{0}},\ldots ,\bar{s_{i}}\right\}$ and a given value in a timestamp $t_{i}$ by $S_{s}\left(t_{i}\right)=s_{i}$. In this work, irradiance on PV surface $G_{I}$, ambient temperature $T_{am}$, PVG output $I_{A}$, PVG output voltage $V_{A}$ and PVG output power generation $P_{A}$ provide five data streams which describe the behavior and energy production of the PV system.

**Figure 5:**
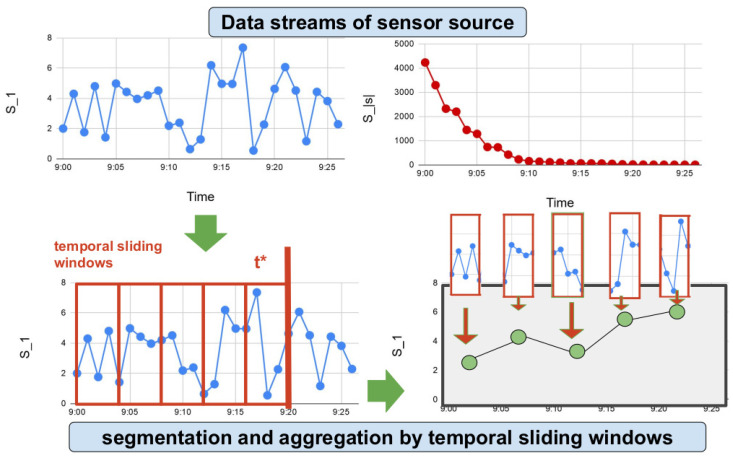
Example of data streams from sensor sources, segmentation and aggregation by temporal sliding windows.

Next, temporal sliding windows, which are defined by the window size of a time interval $W_{w}=\left[W_{w}^{-},W_{w}^{+}\right]$ [45], segment the samples of a sensor stream $\bar{S_{s}}$ and aggregate the values $\bar{s_{i}}$ by a given aggregation function $T_{t}\left(S_{s},W_{w},t^{*}\right)$:

$$T_{t}\left(S_{s},W_{w},t^{*}\right)=\overset{\bar{s_{i}}}{\underset{s_{i}}{⋃}}s_{i},t_{i}\in \left[t^{*}-W_{w}^{-},t^{*}-W_{w}^{+}\right]$$

whose value of aggregation defines a given feature $T_{t}$ of the sensors $S_{s}$ in a current time $t^{*}$. In Figure 5, we describe the segmentation and aggregation by temporal sliding windows in some visual examples of data streams.

### 4.2. Human-Crafted Features and Multiple Temporal Windows for Efficient Nowcasting of Output Power Generation

In this section, we describe human-crafted features based on multiple sliding temporal windows where an expert defines an aggregation function to process sensor streams training a data-driven regressor to compute a feature vector for learning purposes.

Among the broad spectrum of models, we focus on efficient regressor, which enables both learning and evaluating on micro boards in real time under fog computing environments [27]. To this end, we evaluate a human-crafted feature approach [46], where the aggregation functions and multiple windows of different sizes are defined by experts. In concrete terms, we include the following configuration of models:

- Aggregation functions $T_{t}$ based on statistical metrics, such as maximal, minimal, average and standard deviation have been defined in this configuration as they have been demonstrated as relevant features in describing sensor streams [47].
- Segmentation and fusion of temporal information from sensor streams with: (i) single window, (ii) multiple windows [25], and (iii) incremental windows [27] to aggregate data from shorter to longer terms enriching the features of sensor streams. Window size is also defined by human criteria.
- Classification from efficient regressors, with low learning time and training requirements, such as linear regression, k-nearest neighbors (kNN), support vector machines (SVM) and random forest (RF).

Therefore, starting from a set of input sensors $S=\{S_{1},\ldots ,S_{s},\ldots ,S_{\left|S\right|}\}$, a set of window sizes $W=\{W_{1},\ldots ,W_{w},\ldots ,W_{\left|W\right|}\}$ and a set of aggregation functions $T=\{T_{1},\ldots ,T_{t},\ldots ,T_{\left|T\right|}\}$ we define a total number of features |S|×|W|×|T| which describe the sensor streams *S* for each point of time $t^{*}$ [47]. Since our model is based on a data-driven supervised approach, the features which describe the sensor streams are associated for each point of time $t^{*}$ with a target sensor to nowcast $S^{*}$ (not included in the input sensors $S\cap S^{*}=$):

$$T_{1}\left(S_{1},W_{1},t^{*}\right),\ldots ,T_{t}\left(S_{s},W_{w},t^{*}\right),\ldots T_{\left|T\right|}\left(S_{\left|S\right|},W_{\left|W\right|},t^{*}\right)\to S^{*}\left(t^{*}\right)$$

### 4.3. Deep Learning Modeling to Nowcast Output Power Generation

In this section, we describe DL models to nowcast output power generation in a PV device. Contrary to the previous proposal, DL does not require human-crafted features and data pre-processing is applied to compute a homogeneous sequence of data between the different collection rates from raw sensor sources. Here, a minimal signal segmentation is defined by sliding temporal windows of short-term window size, which is related to a minimal temporal granularity Δ. The raw data are averaged ⋃=μ for each short-term temporal window within the segment.

So, we obtain a sequence of data for each sensor source, whose sequence size is the same for all sources $S_{s}$:

$$S^{*}\left(t^{*}\right)\to \{\begin{array}{c} \mu \left(S_{1},\left[0,\Delta \right],t^{*}\right)\to \mu \left(S_{1},\left[\Delta ,2\Delta \right],t^{*}\right),\ldots ,\mu (S_{1},[\Delta |W|-\Delta ,\Delta |W|],t^{*})\\ \ldots \\ \mu \left(S_{\left|S\right|},\left[0,\Delta \right],t^{*}\right)\to \mu \left(S_{\left|S\right|},\left[\Delta ,2\Delta \right],t^{*}\right),\ldots ,\mu (S_{\left|S\right|},[\Delta |W-1|,\Delta |W|],t^{*}) \end{array}$$

which are related to the target sensor to nowcast $S^{*}$ for each current time $t^{*}$ under a sliding window approach.

Once the input and output data from the DL model are defined, in this work, we propose two architectures of DL neural networks to nowcast the output power generation of the PV device, which have been shown as suitable configurations to sequence time series in sliding window approaches [48].

- 2LSTM. Two layers of LSTM which have been previously identified as a suitable configuration to nowcast energy load [49].
- 3CNN+2LSTM. Three layers of CNN are firstly integrated as spatial feature extractors. Next, two layers of LSTM model the temporal dependencies from CNN. The combination of CNN-LSTM hybrid networks has been selected due to providing encouraging results in modeling output power generation [50].

In Table 2, we include the parameters and layers for each proposed model.

## 5. Evaluation

In this section, we present the evaluation of our proposal. First we shall present the experimental setup, then the results obtained and, finally, we will discuss our proposal based on the results presented.

### 5.1. Experimental Setup

In this section, we describe the experimental setup and results of a case study developed in the University of Jaén (Spain), where the Opera Project and PV device were deployed. The IoT module which collected the photovoltaic data in real time within the Opera platform was running from the 9th of June to the 23rd of November 2019, generating data collection over 168 days. The location of the IoT module in the campus of the University of Jaén was (latitude: 37.787253, longitude: −3.776258).

In the experimental setup, five sensors, which were installed in the PV device, collected the following measures: irradiance, ambient temperature, module temperature, output current and output voltage, as described in Section 3. The output power generation to be estimated by the machine learning model was obtained using output current and output voltage according to the following equation: $P=V\dot{I}$.

Both data and learning models are openly available to the scientific community at this GitHub repository: https://github.com/galmonacid/opera/. Below, we detail the configuration and results in nowcasting output power generation by several machine learning models.

- Human-crafted features and multiple temporal windows. We evaluate the nowcasting performance of the following models with human-crafted features and multiple temporal windows and times with the configurations shown below:
  - Linear regression, with intercept = True.
  - kNN (k-Nearest Neighbors), with number of neighbours = 5.
  - SVM (Support Vector Machine), with kernel = polynomial.
  - Random forest, with minimum samples leaf = 1 and minimum samples split = 2.
  For each of these four models, three sliding temporal window configurations were defined and evaluated:
  - *T* = 10 min, one single 10-min temporal window.
  - *T* = 30 min, three 10-min temporal windows.
  - *T* = 90 min, three incremental temporal windows, with a 10-min, 20-min and 60-min window.
- Deep Learning approaches, where we evaluate the performance and learning time of two DL models: 2LSTM and 3CNN+2LSTM, as described in Section 4.3. Concretely, we have evaluated two segmentation configurations: 10 min Δ = 10 m and 5 min Δ = 5 m:
  - Δ = 10 m defined by a 90-minute sequence of data whose sequence length is |W|=9, *W* = {[0 m, 9 m], [10 m, 19 m], …, [80 m, 89 m]}. For Δ = 10 m and |W|=18*W* = {[0 m, 4 m], [5 m, 9 m], …, [85 m, 89 m]} for Δ = 5 m. This configuration generated a total of 24,031 samples for learning purposes.
  - Δ = 5 m, defined by a 90-minute sequence of data whose sequence length is |W|=18, |W|=18*W* = {[0 m, 4 m], [5 m, 9 m], …, [85 m, 89 m]} for Δ = 5 m. This configuration generated a total of 48,062 samples for learning purposes.

In order to nowcast output power generation from the ambient data collected in the PVS, we compared the predicted and ground truth in the tests using 30-fold cross validation. We note the ambient data from photovoltaic sources has been normalized using the max-min method in a previous learning stage.

### 5.2. Results

In this section, we describe the obtained results from the standard analytical method and the machine learning approaches described in the work.

Output power generation was collected by the IoT module representing the ground truth for evaluation purposes. The estimated output power generation for each model was based on data from ambient sensors. The prediction versus the ground truth for the full time-line of tests were compared using Mean Absolute Error (MAE), Root Mean Squared Error (RMSE) and coefficient of determination (R2). With the 30-fold cross validation configuration, we also computed learning time and evaluation time to assess the resource consumption of the models.

First, we evaluated the Araujo model which provides a base performance provided by the standard analytical method. The results from this baseline model are shown in Table 3:

Second, we evaluated one of the data-driven approaches analyzed in this work: regressor models which nowcast energy generation by means of human-crafted features computed from sensor streams. The results are shown in Table 4 in terms of RMSE, MAE and R2 metrics.

Furthermore, in order to evaluate the computational energy consumption, we have included a comparison of learning and evaluation time for the models based on human-crafted features in Table 5.

Third, we evaluated the data-driven approach based on deep learning. To compare the results with Araujo and models based on human-crafted features, we provide the comparison of the performance of DL models in terms of RMSE, MAE and R2 metrics in Table 6 and the learning and evaluation time in Table 7.

Finally, as summary of the results of the different models, in Table 8 we include a comparison between the different approaches: Araujo, the best-performing DL model (3CNN+2LSTM) and the best regressor among human-crafted feature approaches (random forest 90 min).

**Figure 6:**
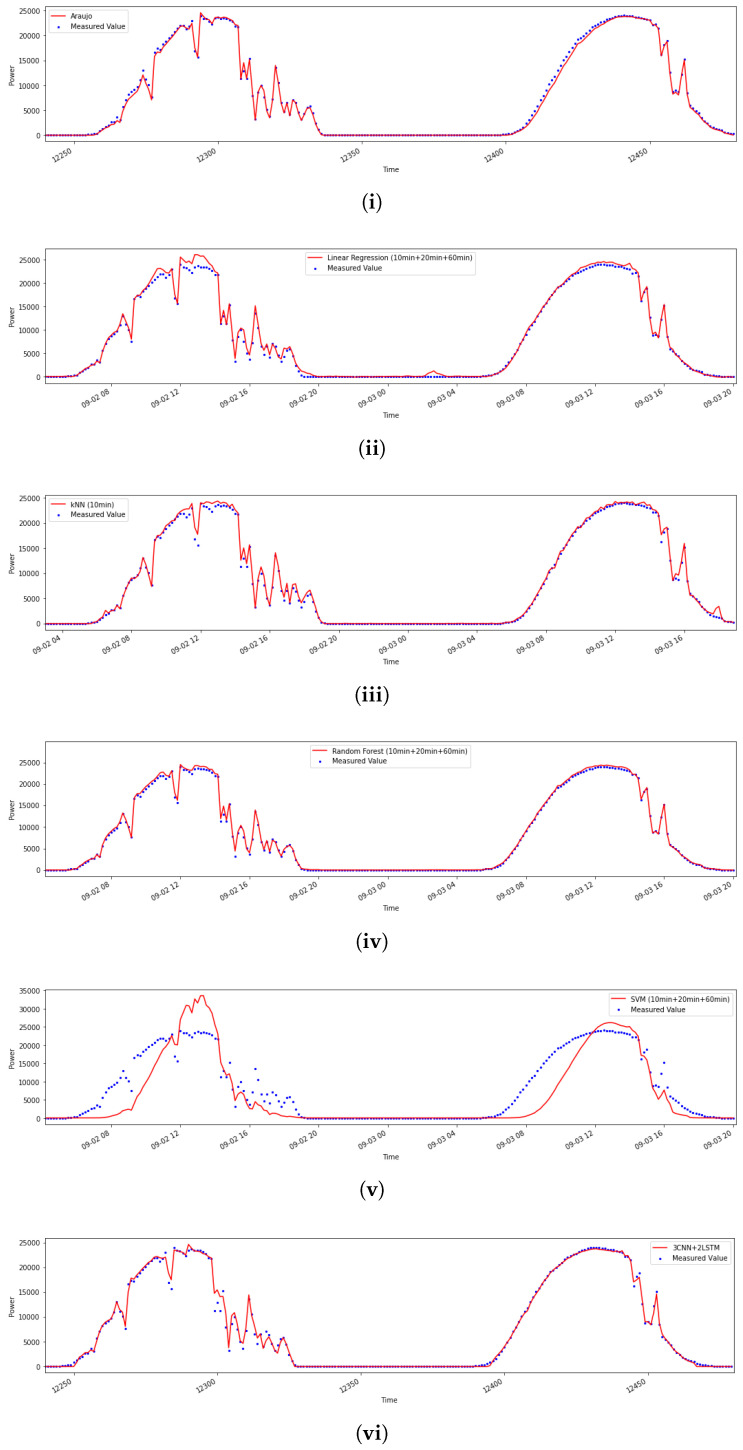
We show 2-day samples of ground truth of output power generation compared with the predictions. From the top to bottom: (**i**) the Araujo model, (**ii**) linear regression, (**iii**) kNN, (**iv**) random forest, (**v**) SVM, (**vi**) 3CNN+2LSTM.

In order to provide a visual representation of the nowcasting of energy consumption, in Figure 6 we show a 2-day sample test comparing measured output power generation with the regressor models.

### 5.3. Discussion

In this work we describe an IoT module for collecting ambient sensor information and output energy consumption from the photovoltaic system deployed under the Opera Project. In order to evaluate the standard behavior of the system and to monitor its performance, we have focused on nowcasting output energy generation from the ambient sensor devices. To this end, two different approaches for machine learning models have been proposed: (i) human-crafted features and multiple temporal windows and (ii) deep learning for automatic feature extraction and learning.

Both approaches present encouraging performance in nowcasting output energy generation in the photovoltaic system based on data collected from ambient sensors; however, we highlight the model based on human-crafted features and multiple temporal windows for its lower learning time and best results. Specifically, we note: (i) the use of multiple imbalanced temporal windows increases nowcasting performance, (ii) random forest is the best regressor and (iii) kNN provides an excellent balance between learning time and results. Moreover, the use of kNN should be highly recommended for nowcasting energy generation in photovoltaic systems using fog-based approaches, where mini boards could perform the data learning in a short time using low computational resources and computational energy consumption.

In the case of DL approaches, the use of CNN+LSTM provides improved nowcasting performance when comparing the results with the Araujo analytical model. This fact is due to the automatic feature extraction generated by CNN, which summarizes the key patterns to nowcast output power generation, providing a remarkable improvement compared with only using LSTM. The performance of the DL model with 10-min segmentation increases compared to 5-min segmentation because short-term segmentation duplicates the number of input variables in the sequence of samples and the higher complexity of data reduces nowcasting performance. However, the human-crafted features model with imbalanced temporal windows has overtaken the performance of DL approaches and the Araujo analytical model, coming out as the leading model according to the results presented in this work.

## 6. Conclusions and Ongoing Works

In this work, an IoT module and data-driven models to nowcast output energy generation integrated in the Opera Digital Platform project have been described. The IoT module is based on Arduino and low-cost sensors which collect ambient and energy data sources in a photovoltaic system. The IoT module has collected the data presented in this work over 24 weeks.

Two approaches based on machine learning have been evaluated: (i) human-crafted features with multiple temporal windows, and (ii) deep learning models. CNN+LSTM, kNN and random forest provide better performance compared with the standard analytical model Araujo. In the case of CNN+LSTM, the advantage of DL is the lack of human intervention in feature definition. The performance of kNN is remarkable, with notably low learning time and providing fog integration capabilities in micro boards. Finally, random forest with incremental temporal windows had the highest performance in terms of error metrics.

A potential advance in this line of work would consist of an in-depth analysis of the diagnosis, typology and fail patterns in PV systems to predict these events by means of machine learning models.

## Figures and Tables

**Table 1:** Variables measured by the data collection system.

Parameter	Symbol	Unit
Irradiance on PV surface	$G_{I}$	W·m${}^{-2}$
Ambient temperature	$T_{am}$	${}^{\circ }$C
PVG output current	$I_{A}$	A
PVG output voltage	$V_{A}$	V
PVG output power generation	$P_{A}$	W

**Table 2:** Configurations of Convolutional Neural Networks.

2LSTM	3CNN+2LSTM
LSTM (32 units)	2 kernels × 16 filters
dropout (0.25)	Re-Lu
LSTM (32 units)	2 kernels × 32 filters
dropout (0.25)	Re-Lu
connected (1 unit)	2 kernels × 64 filters
activation function: Re-Lu	Re-Lu
loss function: MAE	dropout (0.25)
	LSTM (32 units)
	dropout (0.25)
	LSTM (32 units)
	dropout (0.25)
	connected (1 unit)
	activation function: Re-Lu
	loss function: MAE

**Table 3:** Araujo error metrics.

Model	RMSE (W)	MAE (W)	R2
Araujo	641.36	354.81	0.9947

**Table 4:** Error metrics of human-crafted feature models with different sliding windows approaches.

Model	Sliding Window Sizes	RMSE (W)	MAE (W)	R2
Linear Regression	10 min	637.20	425.74	0.9948
	10 min + 10 min + 10 min	590.27	375.11	0.9955
	10 min + 20 min + 60 min	537.37	323.04	0.9963
kNN	10 min	466.76	229.62	0.9972
	10 min + 10 min + 10 min	536.11	249.29	0.9963
	10 min + 20 min + 60 min	528.40	253.13	0.9964
**Random Forest**	10 min	410.44	201.91	0.9978
	10 min + 10 min + 10 min	375.49	183.49	0.9982
	**10 min + 20 min + 60 min**	**360.13**	**173.47**	**0.9983**
SVM	10 min	4474.17	2794.36	0.7421
	10 min + 10 min + 10 min	4593.69	2835.49	0.7281
	10 min + 20 min + 60 min	4410.17	2653.65	0.7493

**Table 5:** Human-crafted feature models time metrics.

Model	Sliding Window Sizes	Learning Time (ms)	Evaluation Time (ms)
Linear Regression	10 min	11.46	2.10
	10 min + 10 min + 10 min	36.67	2.72
	10 min + 20 min + 60 min	37.25	2.81
kNN	10 min	86.52	13.00
	10 min + 10 min + 10 min	213.55	51.50
	10 min + 20 min + 60 min	196.54	52.11
Random Forest	10 min	22,743.35	42.89
	10 min + 10 min + 10 min	69,790.30	45.54
	10 min + 20 min + 60 min	73,499.40	42.03
SVM	10 min	30,912.46	322.88
	10 min + 10 min + 10 min	48,459.79	846.61
	10 min + 20 min + 60 min	49,373.02	857.88

**Table 6:** Error metrics of Deep Learning approaches based on Long Short-Term Memory (LSTM) and Convolutional Neural Network (CNN)+LSTM.

Model	Segmentation	RMSE (W)	MAE (W)	R2
2LSTM	5 min	2393.75	618.70	0.9262
	10 min	706.57	376.76	0.9936
3CNN+2LSTM	5 min	2384.14	583.11	0.9271
	10 min	531.08	274.87	0.9964

**Table 7:** Deep Learning models time metrics.

Model	Segmentation	Learning Time (ms)	Evaluation Time (ms)
2LSTM	10 min	222,657.12	6951.65
3CNN+2LSTM	10 min	197,593.52	5627.01

**Table 8:** Summary of error metrics for best configurations of human-crafted features and DL approaches.

Model	RMSE (W)	MAE (W)	R2
**Araujo**	641.36	354.81	0.9947
**3CNN+2LSTM**	531.08	274.87	0.9964
**Random Forest**	360.13	173.47	0.9983

## Abbreviations

The following abbreviations are used in this manuscript:

FF	Fill Factor
IoT	Internet of Things
LD	linear dichroism
CNN	Convolutional Neural Network
LSTM	Long Short-Term Memory
DL	Deep Learning
O&M	Operation and Maintenance
PV	Photovoltaic
PVG	PV Generator
PVS	PV Systems
STC	Standard test conditions for solar cells
